# Application of Neurokinin-1 Receptor in Targeted Strategies for Glioma Treatment. Part I: Synthesis and Evaluation of Substance P Fragments Labeled with ^99m^Tc and ^177^Lu as Potential Receptor Radiopharmaceuticals

**DOI:** 10.3390/molecules23102542

**Published:** 2018-10-05

**Authors:** Agnieszka Majkowska-Pilip, Przemysław Koźmiński, Anna Wawrzynowska, Tadeusz Budlewski, Bogusław Kostkiewicz, Ewa Gniazdowska

**Affiliations:** 1 Centre of Radiochemistry and Nuclear Chemistry, Institute of Nuclear Chemistry and Technology, Dorodna 16, 03-195 Warsaw, Poland; p.kozminski@ichtj.waw.pl (P.K.); e.gniazdowska@ichtj.waw.pl (E.G.); 2 Warsaw University of Life Sciences, Faculty of Agriculture and Biology, Nowoursynowska 159, 02-776 Warsaw, Poland; anna-rawicz@wp.pl; 3 Radionuclide Therapy Ward, Central Clinical Hospital of the Ministry of the Interior and Administration, Wołoska 137, 02-507 Warsaw, Poland; tbudlewski@gmail.com; 4 Clinical Department of Neurosurgery, Central Clinical Hospital of the Ministry of Interior and Administration, Wołoska 137, 02-507 Warsaw, Poland; boguslaw.kostkiewicz@gmail.com

**Keywords:** gliomas, substance P, NK-1 receptor, ^177^Lu and ^99m^Tc radionuclides

## Abstract

Gliomas, particularly WHO grade IV glioblastoma multiforme, are one of the most common and aggressive primary tumors of the central nervous system. The neuropeptide, substance P (SP), is the physiological ligand of the neurokinin-1 (NK-1) receptor that is consistently overexpressed in glioblastoma cells. The aim of this work was to study physico-chemical and biological properties of different SP analogues labeled with technetium-99m and lutetium-177 radionuclides. The synthesized compounds were characterized in vitro by partition coefficients (log*P*) and their stability was investigated in various physiological solutions. Biological properties (*K_d_,* *B_max_*) were characterized using the U373 MG cell line. The obtained lipophilicity values of the [^99m^Tc]NS_3_/CN-SP and [^177^Lu]DOTA-SP radiobioconjugates were in the range of −0.3 to +0.6 and −2.5 to −5.0, respectively. The studied radiobioconjugates were stable in PBS buffer and CSF, as well as in 10 mM histidine and/or cysteine solutions whereas in human serum showed enzymatic biodegradation. [^177^Lu]DOTA-[Thi^8^,Met(O_2_)^11^]SP(1–11), [^177^Lu]DOTA-SP(4–11) and [^177^Lu]DOTA-[Thi^8^,Met(O_2_)^11^]SP(5–11) radiobioconjugates bound specifically to NK-1 receptors expressed on glioblastoma cells with affinity in the nanomolar range. To conclude, the shorter analogues of SP can be used as vectors, nevertheless they still do not fulfil all requirements for preparations in nuclear medicine.

## 1. Introduction

Gliomas are the most common primary brain tumors in adult patients. The most malignant of them is glioblastoma multiforme (GBM), also called glioblastoma or astrocytoma. Grade IV GBM is a very aggressive primary brain tumor with unknown etiology in most cases [1]. GBM appears to account for over 50% of all brain tumors and more than 20% of all intracranial tumors. The infiltrating character of these tumors (clearly defined margins are absent) and the significant morphological cell heterogeneity (many types of cells are present) of the tumor tissue as well as the protective effect of the blood-brain barrier are the main causes of insufficient treatment with surgery, radiotherapy and chemotherapy. Currently, about 90% of patients have tumor recurrence at the original tumor location or at a distance of two centimeters from the primary tumor. Approximately 50% of people diagnosed with GBM die within one year, while 90% die within three years [2,3]. GBM displays resistance to almost all current anti-cancer approaches: chemo- or radiotherapy, and even to the induction of apoptosis. Median survival with standard-of-care radiation and chemotherapy is 15 months whereas median survival without treatment is only four and a half months. Despite recent advances in cancer treatment, the most invasive types of brain tumors remain inherently difficult to treat and the prognosis for these patients remains extremely poor. Application of one of the most intensively developing new directions in tumor treatment-targeted radionuclide therapy (TRT)—a therapy based on the use of high-affinity biomolecules as carriers (vectors) of therapeutic radionuclides to tumor cells—has created novel opportunities in glioma treatment [4]. A properly chosen vector (in the case of gliomas, the neuropeptide substance P) that has a high affinity for an overexpressed receptor (on the glioma cells, neurokinin-1 receptors) concentrates the therapeutic radionuclide around the target point. This is where the energy of the emitted radiation deposited in tumor tissue selectively destroys tumor cells. Neurokinin-1 (NK-1) receptor is the main receptor of peptides belonging to the tachykinin family [5]. Overexpression of the NK-1 receptor is present in a broad variety of tumors including melanoma, glioma, pancreatic cancer, and cancers of the larynx, stomach and breast. The number of NK-1 receptors expressed on tumor cells is much greater than that on normal human cells and is correlated with the degree of malignancy [5,6,7,8]. Overexpression of NK-1 receptors on tumor cells has allowed use of substance P (SP, the physiological ligand of the NK-1 receptor) in cancer treatment [9,10,11,12]. SP is a neuropeptide containing 11 amino acids (Arg^1^Pro^2^Lys^3^Pro^4^Gln^5^Gln^6^Phe^7^Phe^8^Gly^9^Leu^10^Met^11^) that is widely distributed in the peripheral and central nervous systems. Together with other neurotransmitters, such as serotonin and dopamine, SP acts as a neuromodulator [8]. Interaction of SP with the NK-1 receptor consists of internalization of the peptide into the cell by the clathrin-dependent mechanism, where the endosome (a combination of the peptide and receptor) dissociates due to more acidic conditions. The released receptor comes back to the cell membrane and the absorbed peptide particles couple with the lysosome where they are processed for further application by the cell (transmission of morphological information) [7]. The SP fragment responsible for its affinity towards the NK-1 receptor is a sequence of five amino acids: Phe^7^Phe^8^Gly^9^Leu^10^Met^11^ located at the C-terminus of the peptide [8]. From March 2012, modified SP ([Thi^8^,Met(O_2_)^11^]-SP) labeled with ^213^Bi has been used in medical experiments for GBM treatment at the Department of Nuclear Medicine (Central Clinical Hospital, Warsaw, Poland) in cooperation with the Institute for Transuranium Elements (JRC-ITU, Karlsruhe) [13]. A preparation of [^213^Bi]DOTA-[Thi^8^,Met(O_2_)^11^]-SP is given independent of routine treatments to patients who have been diagnosed with recurrent critically located GBM. It is administered locally into the solid cancer or into the cavity after surgical removal of the tumor. This method delivers a higher dose of the drug into the tumor while avoiding the blood-brain barrier. A notable disadvantage of treatment with [^213^Bi]DOTA-[Thi^8^Met(O_2_)^11^]-SP is its poor migration into the walls of the post-surgical cavity. This makes it difficult to reach and destroy single glioblastoma cancer cells including stem cells.

The aim of this research was to synthesize and study new radiobioconjugates with higher lipophilicity and lower molecular weight, which would exhibit more effective migration into solid tumors or the post-surgical cavity walls. As biomolecules used for synthesis of novel radiobioconjugates, shorter and/or modified fragments of SP have been used. Biological study of novel preparations allows evaluation of the receptor affinity of new radiobioconjugates containing different SP analogues as a vector. It also provides the possibility of applying the SP/NK-1 receptor system to glioblastoma multiforme treatment.

## 2. Results and Discussion

**Figure 1:**
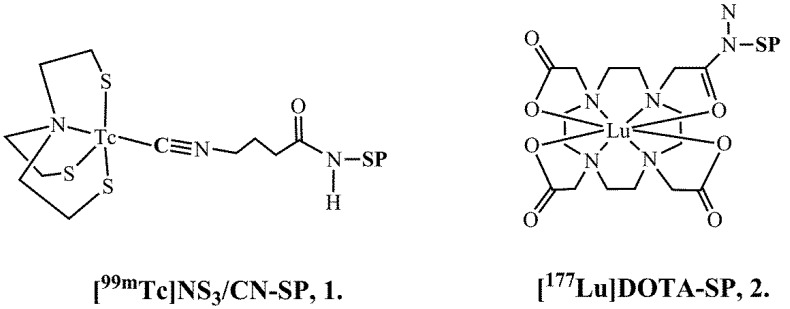
Structure of [^99m^Tc]NS_3_/CN-SP (1) and [^177^Lu]DOTA-SP (2) radiobioconjugates.

In this work, two series of radiobioconjugates have been synthesized and studied (Figure 1). 

**Figure 2:**
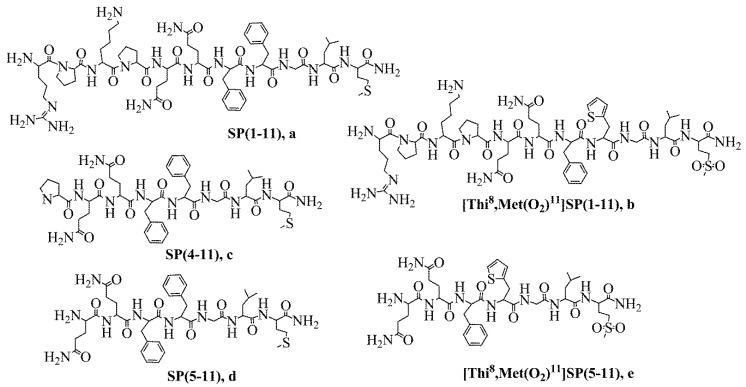
Structure of substance P (SP) peptide and its analogues used in this work.

**Figure 3:**
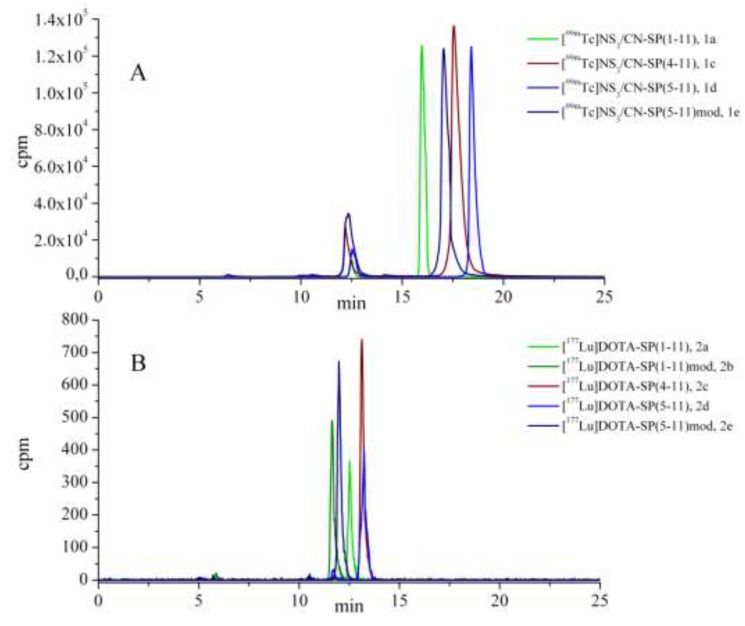
HPLC chromatograms (system 2) of SP analogue labeling reactions with: A: technetium-99m (**1a–e**):, the small peaks at R_T_ = 12.2–12.5 min correspond to the intermediate complex [^99m^Tc](NS_3_) and B: lutetium-177 (**2a–e**).

In the first series ([^99m^Tc]NS_3_-SP, **1**), the biomolecules were labelled with diagnostic radionuclide technetium-99m (emitter γ, t_½_ = 6.01 h, E_max_ = 0.141 MeV) as an analog of therapeutic rhenium-188. Technetium and rhenium are congeners and owing to lanthanide contraction, they have almost identical ionic radii. This indicates that these two elements form analogous complexes (differing only in the metal center) with exactly the same chemical structure and stability. Due to the fact that these species should exhibit the same “in vivo” biological behavior [14,15]. In the second series ([^177^Lu]DOTA-SP, **2**), the biomolecules were labelled with therapeutic radionuclide lutetium-177 (emitter β, t_½_ = 6.71 days, E_max_ = 0.497 MeV). The ^99m^Tc radiobioconjugates contained [^99m^Tc]Tc^3+^ cation coordinated by the tetradentate NS_3_ tripodal chelator (tris(2-mercaptoethyl)-amine; 2,2’,2’’-nitrilotriethanethiol) and a monodentate isocyanide species CN-BFCA (bifunctional coupling agent, isocyanobutyric acid succinimidyl ester). These technetium-99m complexes are extremely stable not only in the thermodynamic sense, but also towards ligand exchange in vivo [16,17]. In the case of ^177^Lu radiobioconjugates, the [^177^Lu]Lu^3+^ cation was coordinated by the macrocyclic ligand DOTA (used in the syntheses in the active ester form, mono-N-hydroxysuccinimide, DOTA-NHS). In both series, the bifunctional coupling agent (CN-BFCA or DOTA-NHS) was coupled with the selected SP-analogue beforehand. The labeled SP analogues (Figure 2) were: SP(1–11) and its modified form [Thi^8^,Met(O_2_)^11^]SP(1–11), SP(4–11), and SP(5–11) and its modified form [Thi^8^,Met(O_2_)^11^]SP(5–11). All analogues contained a sequence of five amino acids at the C-terminus of the peptide, responsible for their affinity towards the NK-1 receptor [8]. The HPLC chromatograms of labeling reactions with technetium-99m (**1a–e**) and lutetium-177 (**2a–e**) are presented in Figure 3. The small peaks on the HPLC chromatograms of the SP analogue labeling reactions with technetium-99m (**1a–e**) recorded at R_T_ = 12.2–12.5 min (Figure 3A) corresponded to the intermediate complex [^99m^Tc](NS_3_) [17].

For all obtained [^99m^Tc]NS_3_/CN-SP (**1a–e**) and [^177^Lu]DOTA-SP (**2a–e**) radiobioconjugates, studies of their physico-chemical properties (so called ‘challenge experiments’, which are important from the view-point of receptor radiopharmaceuticals) were performed [18]. Stability of the radiobioconjugates was investigated in various physiological solutions, namely PBS, histidine and cysteine. The experimental results, verified by HPLC (high-performance liquid chromatography) and ITLC (instant thin layer chromatography) methods, showed that in 10^−2^ M PBS solution and 10^−3^ M cysteine and/or histidine solutions (containing a 1000 times excess of strongly competing natural ligands compared to the concentration of the tested radiobioconjugate) all studied compounds were stable. On the recorded HPLC chromatograms (after 24 h and 15 days of incubation in the case of **1a–e** and **2a–e** compounds, respectively) only single peaks were observed with R_T_ corresponding to R_T_ values of the tested radiobioconjugates. Thus, we consider that the studied radiobioconjugates do not undergo ligand exchange reactions with amino acids or other ligands containing strongly reactive SH or NH groups. The lipophilicity parameters (log*P*) for all radiobioconjugates (expressed as the logarithms of their partition coefficients, P) determined in a biphasic mixture of *n*-octanol and PBS (pH 7.40) are presented in Table 1 and Table 2.

**Figure 4:**
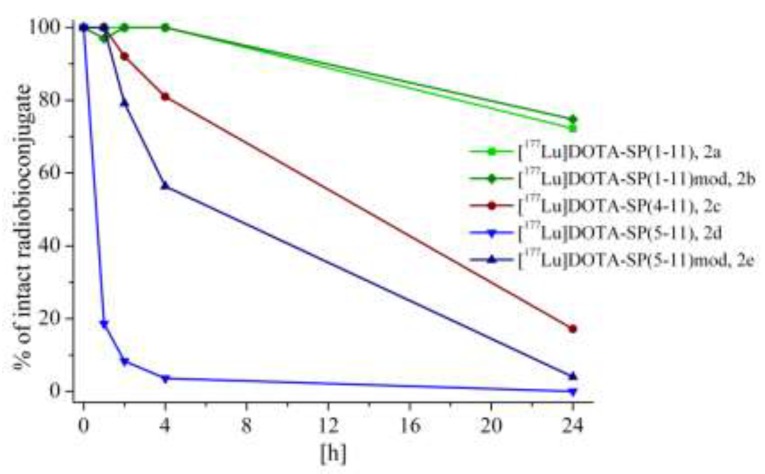
Stability studies of [^177^Lu]DOTA-SP, **2a–e**, radiobioconjugates in human serum.

The lipophilicity value of a radiobioconjugate very strongly depends on the hydrophilic-hydrophobic properties of the radionuclide complex and the nature of the biomolecule used as a vector. In the series of [^99m^Tc]NS_3_/CN-SP radiobioconjugates, the lipophilicity values were several orders of magnitude higher than those in the series of [^177^Lu]DOTA-SP radiobioconjugates. This was due to the high hydrophobic character of the NS_3_ ligand and the hydrophilic character of the macrocyclic ligand DOTA, respectively. Further, analysis of determined values showed that radiobioconjugates contain shorter SP fragments. Therefore, they are of lower molecular weight, and characterized by higher lipophilicity parameters. In contrast, replacement of amino acids in positions 8 and 11 (Phe and Met by Thi and Met(O_2_), respectively) to increase the half-life of the peptide resulted in a decrease in lipophilicity. In both series, despite the very different ranges of log*P* values from about −0.3 to +0.6 and −2.5 to −5 for **1a–e** and **2a–e** radiobioconjugates, respectively, we observed the same trend in changes of radiobioconjugate lipophilicity values. This remained in good accordance with the direction of the log*P* changes of the SP fragments (theoretically calculated by the program, MarvinSketch) used for radiobioconjugate synthesis. Bearing in mind the potential application of new radiobioconjugates based on SP fragments in GBM treatment (containing diagnostic or therapeutic radionuclides, e.g., ^68^Ga, ^213^Bi or ^225^Ac, chelated by the macrocyclic ligand, DOTA), stability studies for [^177^Lu]DOTA-SP radiobioconjugates in cerebrospinal fluid (CSF) and human serum (HS) were also performed. Stability studies in CSF showed that **2a–e** radiobioconjugates were stable in this medium with no enzymatic biodegradation of [^177^Lu]DOTA-SP radiobioconjugates. More specifically, there was no enzymatic biodegradation of biomolecules (vectors) observed. The percentage of the **2a–e** radiobioconjugate bound by CSF components was in the range of 1–4%, while about 96% of the studied compounds remained in the liquid phase in unchanged form. Similar to previous ‘challenge experiment’ studies, the HPLC chromatograms recorded after different time intervals of incubation (up to 15 days) showed the existence of one main radioactive species in the solution, with the retention time characteristic for the studied radiobioconjugate. However, stability studies of the **2a–e** radiobioconjugates in HS showed an unacceptable compound stability in these conditions (Figure 4).

**Figure 5:**
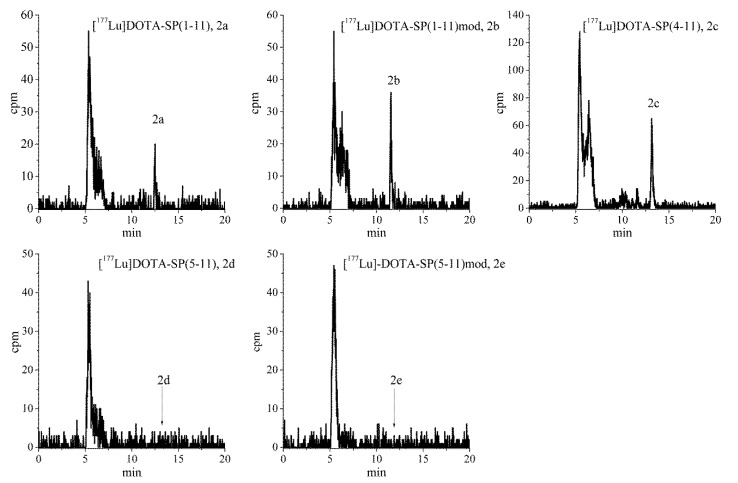
HPLC chromatograms of **2a–e** radiobioconjugates after incubation in human serum at 37 °C for 24 h.

**Figure 6:**
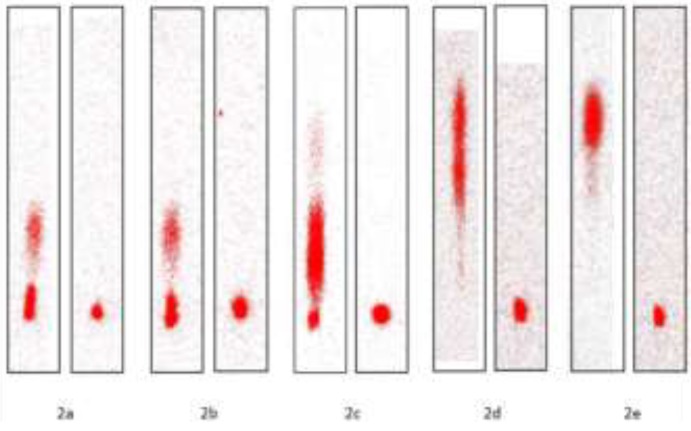
ITLC analyses of **2a–e** radiobioconjugates after incubation in human serum at 37°C for 24 h.

HPLC chromatograms of the liquid phases (after precipitation and separation of the radiobioconjugate bound by protein components present in HS) showed new peaks while the peak corresponding to the studied radiobioconjugate disappeared (Figure 5). Similarly, we observed more than one stain on the ITLC strips (Figure 6).

The appearance of new peaks on HPLC chromatograms indicated enzymatic biodegradation of SP, as well as all its analogues (protein content in HS is about 200-fold higher than in CSF) [19]. The results also showed that enzymatic biodegradation of radiobioconjugates containing shorter SP fragments occurred faster than with radiobioconjugates containing SP molecules with the full amino acid sequence (Table 2, Figure 5). The enzyme most specific for degrading SP is neutral endopeptidase (NEP) [20]. NEP cleaves SP into shorter fragments [SP(1–11), SP(4–11), SP(5–11), SP(6–11) and SP(8–11)], which generally have a higher formation rate. The lower stability of radiobioconjugates containing shorter SP fragments is probably caused by easier matching of the smaller peptide to the enzyme cellular matrix (more accurate adjustment of the substrate to the active sites of the enzyme). The stability study results for the **2a–e** radiobioconjugates in HS assessed by HPLC method corresponded very well with the results obtained using the ITLC method (Figure 6). For each of the **2a–e** radiobioconjugates, two TLC strips were presented, showing the location of spots after incubation (left-hand strip) and, as a blank, without incubation in HS (right-hand strip). In the case of **2a**, **2b** and **2c** radiobioconjugates (within 24 h of incubation), the enzymatic biomolecule biodegradation was only partial. However, for the **2d** and **2e** compounds (within the same incubation time), virtually all of the radiobiomolecule that was deposited onto the strip was degraded enzymatically (there were no stains at the origin on the strip). During incubation in HS, the proportion of the **2a–e** compounds bound by serum protein components was in the range of 8–11%. The stability data presented in Table 2, and Figure 5 and Figure 6, shows that modification of the SP(5–11) fragment in positions 8 and 11 (replacement of Phe and Met by Thi and Met(O_2_), respectively) caused a significant improvement in radiobioconjugate stability in HS (stability of **2e** was significantly higher than that of **2d**). The same modification of the entire peptide, SP(1–11), had no effect on radiobioconjugate stability (the stability of **2b** and **2a** radiobioconjugates in HS were comparable). This led us to conclude that the radiobioconjugate based on the fragment SP(4–11), with prior modification in an analogous way (i.e., [^177^Lu]DOTA-[Thi^8^,Met(O_2_)^11^]SP(4–11) compound), would also be characterized by better stability in HS than the compound **2c.** However, simultaneously the lipophilicity parameter of this compound would become slightly lower.

**Figure 7:**
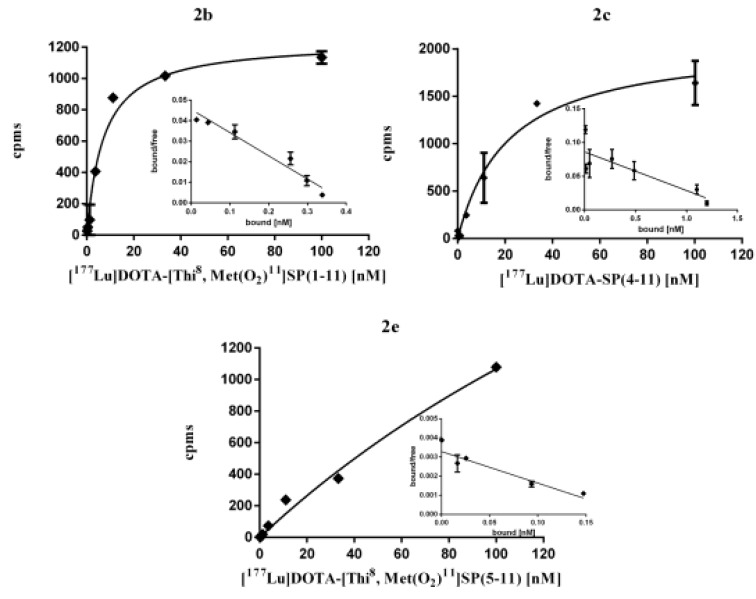
Specific binding of **2b**, **2c** and **2e** radiobioconjugates performed on the U373 MG cell line expressing the NK-1 receptor.

From the **2a–e** compound series, the radiobioconjugates **2c** and **2e** were selected for biological studies due to their relatively low molecular weight and high lipophilicity. The stability of both radiobioconjugates in HS was found to be unsatisfactory. However, for GBM treatment these preparations are administered locally into the solid cancer or into the cavity after surgical tumor removal (where the content of serum components is rather low). Therefore, relatively low radiobioconjugate stability in HS does not appear to be a crucial parameter for its potential application. Biological studies were also performed for the **2b** compound—this radiobioconjugate contains, as a vector, the same biomolecule [modified native SP peptide [Thi^8^,Met(O_2_)^11^]SP(1–11)] as the preparation of [^213^Bi/^225^Ac]DOTA-[Thi^8^,Met(O_2_)^11^]-SP currently used in GBM therapy. As values obtained experimentally in biological studies are determined in different conditions and laboratories, they cannot be considered ‘absolute’ and their comparison can only be qualitative. Therefore, the biological properties of the new **2c** and **2e** radiobioconjugates were compared with the properties of compound **2b** used in our studies as a reference. The biological properties of **2b**, **2c** and **2e** radiobioconjugates were characterized in vitro by investigation of their affinity to the NK-1 receptor using the U373 MG cell line (Uppsala, human glioblastoma astrocytoma derived from brain malignant tumor). Biodistribution studies in vivo, usually performed in biological tests for novel preparations, were not planned for **2b**, **2c** and **2e** radiobioconjugates (containing as a vector SP peptide or its analogues) for two reasons. First, in GBM targeted therapy, the radiobioconjugates are administered directly into the solid cancer or into the postsurgical cavity. Second, SP is a potent vasodilator, which results in a reduction in blood pressure. To characterize the receptor-binding properties of SP radiobioconjugates, saturation binding experiments on the U373 MG cells were performed (Figure 7), with determination of *K_d_* and *B_ma_*_x_ values (Table 3). These results demonstrated that both newly synthesized **2c** and **2e** radiobioconjugates bind specifically to NK-1 receptors expressed on GBM cells with high affinity in the nanomolar range. Nevertheless, the receptor affinity of the **2c** radiobioconjugate (*K_d_* = 13.3 ± 1.6 nM) to NK-1 receptor was higher (a lower *K_d_* value corresponds to higher affinity) than compound **2e** (*K_d_* = 48.7 ± 5.1 nM) and very close to the *K_d_* value of **2b** (*K_d_* = 11.1 ± 2.7 nM). Our results showed that new radiobioconjugates, based on the modified SP(1–11) analogues, e.g., SP(4–11) and [Thi^8^,Met(O_2_)^11^]SP(5–11), exhibited good affinity for glioblastoma cancer cells. The obtained *K_d_* values were similar to those found for [^225^Ac]DOTA-[Thi^8^,Met(O_2_)^11^]SP(1–11) and [^90^Y/^177^Lu]DOTAGA-[Thi^8^,Met(O_2_)^11^]SP(1–11) despite the studies on these compounds being carried out on different cell lines, e.g., U251, LN319 and T98G [21,22,23].

## 3. Materials and Methods

### 3.1 Materials

The peptides SP(1–11), SP(4–11) and SP(5–11) were purchased from GeneCust Europe (Ellange, Luxembourg). [Thi^8^,Met(O_2_)^11^]SP(5–11) was synthesized on request at the Institute of Biochemistry and Biophysics Polish Academy of Science (Warsaw, Poland).

The tetradentate NS_3_ ligand (2,2’,2’’-nitrilotriethanethiol) was prepared by reaction of tris(2-chloroethyl)amine hydrochloride with potassium thioacetate followed by reduction with LiAlH_4_ [24]. The substrates for the synthesis of NS_3_ ligand were benzyl bromide (Merck, Darmstadt, Germany), ethylene sulphide (Merck, Darmstadt, Germany) and S-benzyl-L-cysteine (Fluka, Mexico City, Mexico) compounds. The final product was precipitated as the oxalate salt and applied as such in further reactions.

The aliphatic linker, bifunctional ligand CN-BFCA (isocyanobutyric acid succinimidyl ester), was synthesized according to a procedure described elsewhere [25]. For the CN-BFCA synthesis, the following compounds (Sigma-Aldrich) were used: 4-aminobutanoic acid, acetic anhydride, formic acid, N-hydroxysuccinimide and triphenylphosphine. DOTA-NHS (1,4,7,10-tetraazacyclododecane-1,4,7,10-tetraacetic acid mono-N-hydroxysuccinimide ester) was a commercially available product, CheMatech (Dijon, France). DOTA-SP(1–11) and DOTA-[Thi^8^,Met(O_2_)^11^]SP(1–11) conjugates were purchased from CASLO ApS (Kongens Lyngby, Denmark) and Bachem (Bubendorf, Switzerland), respectively.

^99m^Tc radionuclide was obtained in a saline solution from the portable ^99^Mo/^99m^Tc generator (purchased from Radioisotope Centre Polatom, Otwock, Poland) in the form of pertechnetate ion with sodium as the counterbalancing cation (Na[^99m^Tc]O_4_). ^177^Lu radionuclide (radiochemical purity >99.9%) was purchased from the Radioisotope Centre Polatom in the form of 0.04 M HCl solution of lutetium chloride ([^177^Lu]LuCl_3_). All other chemical reagents used for the syntheses and HPLC analyses were purchased from Polish Chemical Reagents S.A. as pure p.a. and used without further purification. Deionized water was prepared in a Hydrolab water purification system (Hydrolab, Straszyn, Poland).

For TLC analysis were used: RP-18 (Merck, Darmstadt, Germany) and iTLC-SG (Agilent, Santa Clara, CA, USA) strips as well as the mixture of H_2_O/ACN, 1:1, *v/v* and 0.05 M sodium citrate solution as developing solvents.

The glioblastoma cell line, U373 MG (Uppsala cell line), was purchased from Sigma-Aldrich. 

Eagle's Minimum Essential Medium (EMEM) was obtained from LGC Standards SP. z o. o. (Dziekanów Leśny, Poland). Conditions of HPLC system: Jupiter Proteo semi-preparative column (4 µm, 90 Å, 250×10 mm; Phenomenex, Torrance, CA, USA), UV/Vis detection at 220 nm in system 1 or γ detection in system 2; elution conditions: solvent A—Water with 0.1% TFA (*v/v*); solvent B—Acetonitrile with 0.1% TFA (*v/v*); gradient: 0–20 min 20 to 80% of B, 20–35 min 80% solvent B; 2 mL/min.

### 3.2 Syntheses

**Scheme 1:**
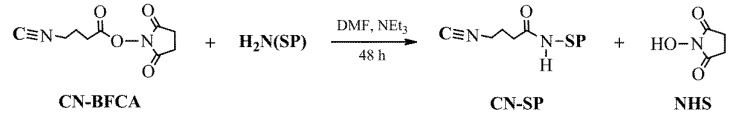
Coupling reaction of CN-BFCA with SP fragments.

**Figure 8:**
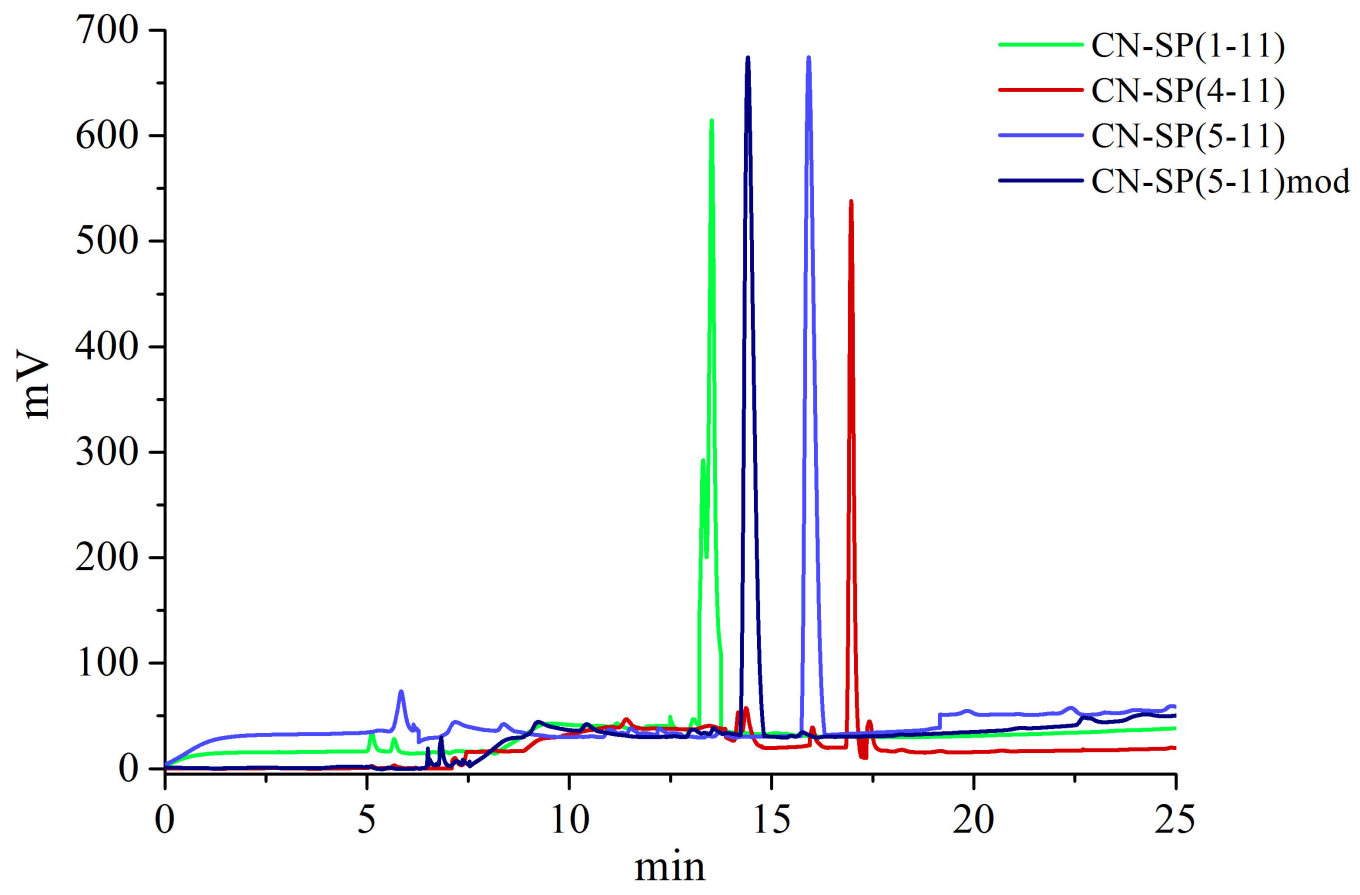
HPLC chromatograms (system 1) of purified CN-SP(1–11), CN-SP(4–11), CN-SP(5–11) and CN-[Thi^8^,Met(O_2_)^11^]SP(5–11) bioconjugates.

The coupling reactions between CN-BFCA (isocyanobutyric acid succinimidyl ester) and four SP analogues [SP(1–11), SP(4–11), SP(5–11) and [Thi^8^,Met(O_2_)^11^]SP(5–11)] were performed in DMF, at 50 °C and in the presence of Et_3_N (Scheme 1). The molar ratio of the reagents used in the coupling reactions was approximately 1.2:1:4, respectively. Crude CN-SP products (Figure 8) were purified on a semi-preparative HPLC column (system 1; Phenomenex, Torrance, CA, USA) and lyophilized (yield ≈85–95%).

MS of CN-SP(1–11): *m/z*: calc. 1441.77; found 1442.33 [M+H^+^]
MS of CN-SP(4–11): *m/z*: calc. 1061.26; found 1062.11 [M+H^+^]
MS of CN-SP(5–11): *m/z*: calc. 964.17; found 965.49 [M+H^+^]
MS of CN-[Thi^8^,Met(O_2_)^11^]SP(5–11): *m/z*: calc. 1003.21; found 1004.41 [M+H^+^]

**Scheme 2:**
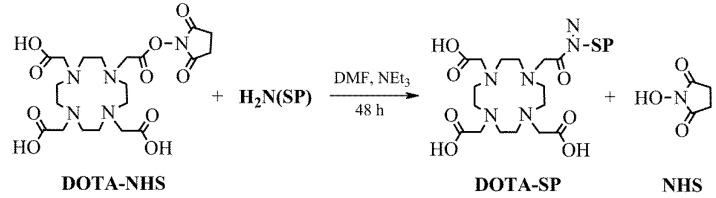
Coupling reaction of DOTA-NHS with SP fragments.

**Figure 9:**
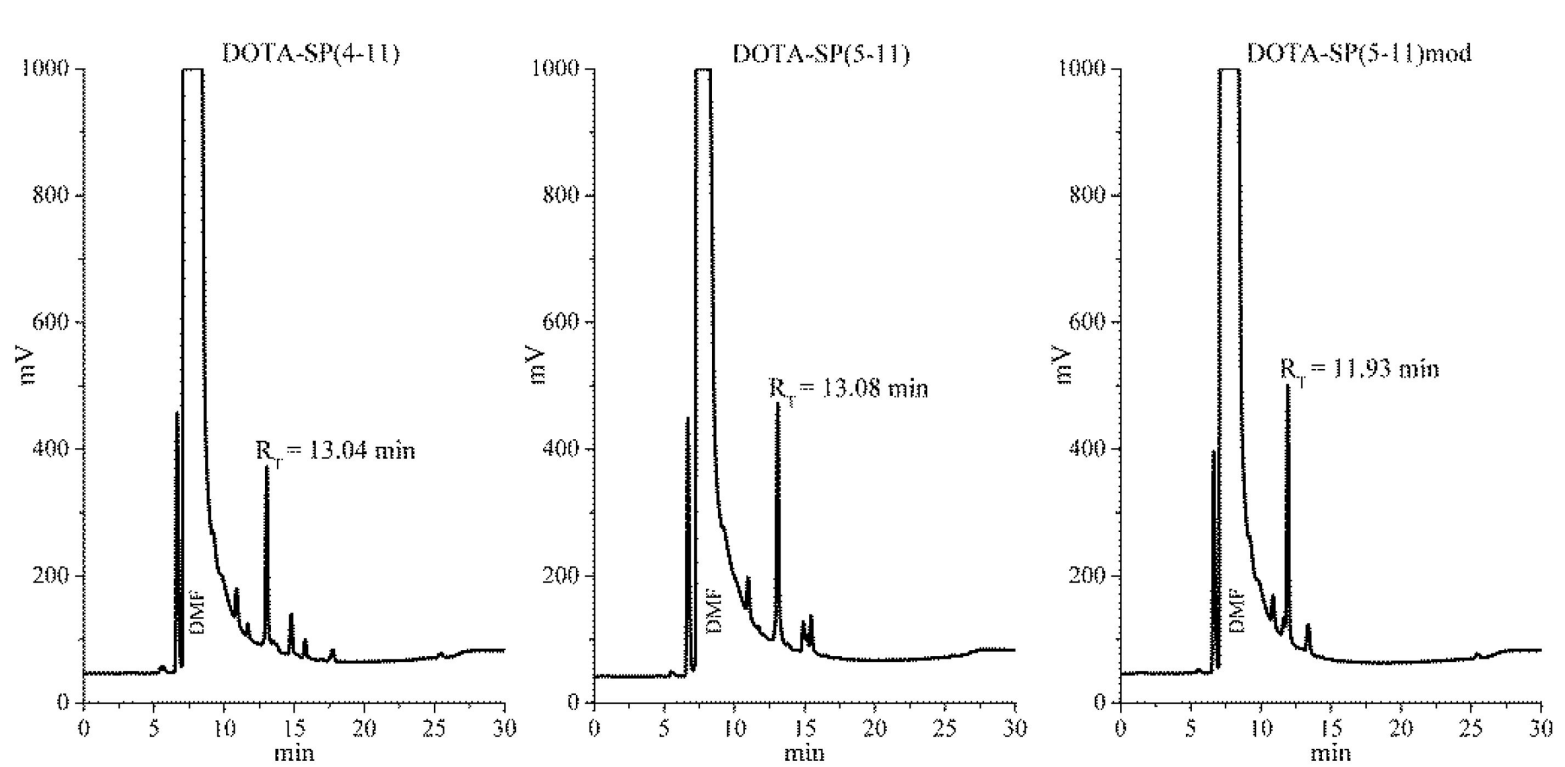
HPLC chromatograms (system 1) of reaction mixtures of DOTA-SP(4–11), DOTA-SP(5–11) and DOTA-[Thi^8^,Met(O_2_)^11^]SP(5–11) bioconjugates.

The coupling reactions between DOTA-NHS (1,4,7,10-tetraazacyclododecane-1,4,7,10-tetraacetic acid mono-N-hydroxysuccinimidyl ester) and three shorter SP analogues [SP(4–11), SP(5–11), [Thi^8^,Met(O_2_)^11^]SP(5–11)] were performed in DMF, at 50°C and in the presence of Et_3_N (Scheme 2). The molar ratio of the reagents used in the coupling reactions was approximately 1:1:4, respectively. Crude DOTA-SP products (Figure 9) were purified on a semi-preparative HPLC column (system 1) and lyophilized (yield ≈80–85%).

A wide peak located at R_T_ 6.7–9 min visible in each chromatogram corresponds to the DMF solvent used in the coupling reactions. This peak covers small peak corresponding to the reaction by product NHS (R_T_ = 6.8 min), while the small peak at R_T_ = 6.6 min corresponds to the reaction substrate DOTA-NHS.

MS of DOTA-SP(4–11): *m/z*: calc. 1351.66; found 1352.88 [M+H^+^]
MS of DOTA-SP(5–11): *m/z*: calc. 1254.61; found 1255.51 [M+H^+^]
MS of DOTA-[Thi^8^,Met(O_2_)^11^]SP(5–11): *m/z*: calc. 1292.55; found 1293.78 [M+H^+^]

### 3.3 Labeling of SP Fragments with ^99m^Tc and ^177^Lu Radionuclides

**Scheme 3:**
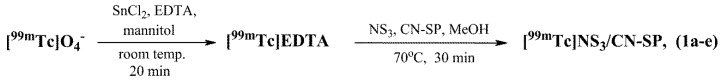
Two-step synthesis of [^99m^Tc]NS_3_/CN-SP radiobioconjugates in no-carrier-added scale.

The [^99m^Tc]NS_3_/CN-SP radiobioconjugates (**1a–e**) were synthesized in two steps (Scheme 3). In the first step, 1 mL of eluate from the [^99^Mo]/[^99m^Tc] generator (100–200 MBq) was added to a kit formulation containing 1 mg of Na_2_EDTA, 5 mg of mannitol and 0.08 mg of SnCl_2_ in freeze-dried form under nitrogen. The mixture was allowed to stand at room temperature for 20 min. The radiochemical purity of the intermediate [^99m^Tc]EDTA/mannitol compound was checked by HPLC and TLC methods. In the second step the [^99m^Tc]EDTA/mannitol compound reacted with 300 µg of the NS_3_ ligand and with about 50 µg of the isocyanide-modified peptide, CN-SP. The reaction progress and radiochemical purity were controlled by HPLC (system 2).

**Scheme 4:**
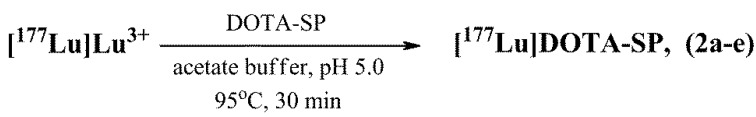
Synthesis of [^177^Lu]DOTA-SP radiobioconjugates.

HPLC chromatograms (system 2) of the reaction mixtures of SP analogue labeling reactions with technetium-99m (for **1a–e** radiobioconjugates) were presented earlier in Figure 3A. The R_f_ (retention factor) values of **1a–e** radiobioconjugates, obtained using the ITLC method (RP-18, Merck, strips and mixture of H_2_O/ACN, 1:1, *v/v*, as developing solvent), were about 0.6. For each radiobioconjugate, we observed only one stain on the strip. The [^177^Lu]DOTA-SP (**2a–e**) radiobioconjugates were synthesized according to the following procedure: to the vial containing 15 nmol of lyophilized DOTA-SP dissolved in 300 µL of 0.4 M acetate buffer (pH 5.0) the solution of [^177^Lu]Cl_3_ (in 0.04 M HCl, 5–10 MBq) was added (Scheme 4). The reaction mixture was heated for 30 min at 95 °C and the reaction progress was checked by ITLC using iTLC-SG strips (Agilent, Santa Clara, CA, USA) and 0.05 M sodium citrate as a developing solvent. Additionally, product efficiency was confirmed by HPLC (system 2). 

HPLC chromatograms (system 2) of the reaction mixtures of the SP analogue labeling reactions with lutetium-177 (for **2a–e** radiobioconjugates) were presented earlier in Figure 3B. The R_f_ values of **2a–e** radiobioconjugates obtained in the ITLC method were about 0.1. The spots did not migrate with the mobile phase and stayed at the origin. All [^99m^Tc]NS_3_/CN-SP and [^177^Lu]DOTA-SP radiobioconjugates were formed with a yield in the range 96–98% and purity higher than 98%.

### 3.4 Physico-Chemical Studies of Radiobioconjugates

All radioconjugates were purified on a semi-preparative column (HPLC method) before using them for physico-chemical and biological tests. Stability tests (so called “challenge experiments”) of **1a–e** and **2a–e** radiobioconjugates were carried out in solutions imitating physiological body fluids, such as 10^−2^ M phosphate buffered saline (PBS), 10^-3^ M cysteine and 10^–3^ M histidine. The radiobioconjugates isolated from the reaction mixture (semi-preparative HPLC method, system 2) were incubated in the solutions at 37 °C for 24 h. At selected time intervals (1, 2, 4 and 24 h), the samples of the incubated solutions were taken and analyzed by TLC and/or HPLC method (system 2). The presence of only one stain on the TLC strip or one peak on an HPLC chromatogram was proof of radiobioconjugate stability.

Stability tests of **2a–e** compounds in CSF and HS were performed similarly to the previous one. The solution (0.1 mL) of isolated **2a–e** radiobioconjugate in 0.1 M PBS buffer (pH 7.4), was added to 0.9 mL of CSF or HS and incubated at 37 °C. After specific time intervals (1, 2, 4 and 24 h), a small sample (0.1–0.2 mL) of the mixture was taken, mixed with ethanol (0.3–0.5 mL) and shaken vigorously to precipitate protein components as well as part of the tested radiobioconjugate bound to protein components. The sample was then centrifuged (14,000 rpm, 5 min) and the supernatant separated. The radioactivities of both supernatant and precipitate were measured using the well-type NaI(Tl) detector (Institute of Nuclear Chemistry and Technology, Warsaw, Poland). Finally, the supernatant fraction was analyzed by HPLC to verify that the studied radiobioconjugate still existed in its intact form.

The lipophilicity (log*P*) of **1a–e** and **2a–e** radiobioconjugates was characterized as the logarithm of their partition coefficients (*P*) determined according to the standard procedure used in studies of biological compounds. The partition coefficient was measured for each compound in a biphasic system of *n*-octanol/PBS (pH 7.4). The biphasic mixture was shaken (vortex, 1 min) and then centrifuged (14,000 rpm, 5 min) before the organic (*n*-octanol) and aqueous (PBS, pH 7.4) phases were separated. The activity of each phase (corresponding to the content of radiobioconjugate) was determined by measuring γ-radiation, using a well-type NaI(Tl) detector. P was calculated as the ratio of activity in the organic versus aqueous phases (as an average value from at least three independent measurements). Immediately after the γ-radiation measurements, the aqueous phase was analyzed by HPLC (system 2) to determine whether the tested radiobioconjugate existed in the liquid phase in an unchanged form.

### 3.5 Biological Studies of Radiobioconjugates

#### 3.5.1 Cell-Binding Studies

To determine the affinity of selected radiobioconjugates: [^177^Lu]DOTA-[Thi^8^,Met(O_2_)^11^]SP(1–11), **2b**, [^177^Lu]DOTA-SP(4–11), **2c**, and [^177^Lu]DOTA-[Thi^8^,Met(O_2_)^11^]SP(5–11), **2e**, to the NK-1 receptors expressed on glioblastoma cells (U373 MG), saturation binding studies were performed. One day before the experiment, 8 × 10^5^ cells/well were seeded in six-well plates. To determine total binding, different concentrations of peptides (15.3 pM to 100 nM) were added to the cells and incubated at 37 °C for 1.5 h. Cellular uptake was stopped by aspirating the reaction supernatant, followed by washing twice with 1 mL of cold PBS. Finally, the cells were lysed using 1 M NaOH to detach them from the well plates. To determine nonspecific binding, the cells were incubated with 1000-fold excess of unlabelled peptides. The radioactivities of collected samples with cell pellets and supernatants were measured using Perkin Elmer automatic γ-counter. The specific binding was obtained by subtracting nonspecific binding from total binding. For estimating the receptor density (*B_max_*) and the dissociation constant (*K_d_*) of the receptor for the studied radiobioconjugates, the Scatchard analysis method was used. Results are means ±SD of three individual experiments.

## 4. Conclusions

The obtained results showed that the lipophilicity of the radiobioconjugates strongly depended on the hydrophilic-hydrophobic properties of the radionuclide complex and the structure of the SP fragment. Radiobioconjugates containing shorter SP fragments were characterized by a lower molecular weight and higher lipophilicity, which allows more effective migration into GBM tissue or into the post-surgery cavity walls. Further, replacement of Phe by Thi in position 8 and Met by its oxidized form, MetO_2_, in position 11 (to increase the half-life of the peptide) resulted in a lipophilicity decrease.

Stability studies of [^99m^Tc]NS_3_/CN-SP and [^177^Lu]DOTA-SP radiobioconjugates showed that all preparations were stable in different physiological solutions (PBS, histidine and cysteine). The [^177^Lu]DOTA-SP radiobioconjugate was also stable in CSF, but not sufficiently stable in HS. Radiobioconjugates containing shorter SP fragments were characterized by lower stability in HS due to the faster enzymatic biodegradation of biomolecules. We performed biological studies on the two newly synthesized and tested radiobioconjugates, [^177^Lu]DOTA-SP(4–11) and [^177^Lu]DOTA-[Thi^8^,Met(O_2_)^11^]SP(5–11), and showed that they specifically bound with high affinity (in the nanomolar range) to NK-1 receptors expressed on the U373 MG cell line (their *K_d_* values were close to the *K_d_* values of preparations currently used in medical experiments for GBM treatment). At the same time, both formulations had a lower molecular weight and higher lipophilicity compared to preparations used currently.

To summarize, the SP analogues tested in this work can be used (taking into account the lipophilicity and receptor affinity of [^177^Lu]DOTA-SP radiobioconjugates) as biologically active molecules (potential vectors) able to lead the diagnostic or therapeutic radionuclide to NK-1 receptors overexpressing on glioblastoma cells. The unsatisfactory stability of [^177^Lu]DOTA-SP radiobioconjugates tested in HS may be an additional reason (apart from the low lipophilicity and high molecular weight of the preparations) for inefficient migration of the receptor radiopharmaceuticals into the walls of post-surgery cavity. After enzymatic vector biodegradation, the therapeutic radionuclides remain at the administration site, so they do not reach and destroy single glioblastoma cancer cells (including stem cells) placed in the tissues around the post-surgery cavity. On the other hand, the unsatisfactory stability of [^177^Lu]DOTA-SP radiobioconjugates in HS does not seem to be a disqualifying parameter because the content of serum components in the post-surgery cavity is rather low. However, it is possible that a hematoma will form in the post-surgical cavity increasing local concentrations of serum components. Application of receptor radiopharmaceuticals, both diagnostic and therapeutic, in accordance with the rules of the use of preparations in nuclear medicine, requires individual consideration (personalized medicine) for each patient [26]. Imaging techniques such as magnetic resonance imaging (MRI) or computed tomography (CT) performed before application of radiobioconjugates allows assessment of the advisability and safety of selected treatment strategies. Our overall conclusion is that potential receptor radiopharmaceuticals based on SP analogues do not fulfil all requirements for preparations used in nuclear medicine. As a result, our current studies are focused on the search for other vectors including peptidic and nonpeptidic antagonists of the NK-1 receptor. This work is in progress and obtained results will be the subject of the new work “Application of neurokinin-1 receptor in therapeutic targeted strategies for glioma treatment, Part II.”

## Figures and Tables

**Table 1:** Physico-chemical properties of SP fragments used in the work and [^99m^Tc]NS_3_/CN-SP radiobioconjugates (R_T_ values were determined using HPLC (system 2)).

Compounds	R_T_ [min]	log*P*
SP(1–11)	12.28	−5.8*
[Thi^8^,Met(O_2_)^11^]SP(1–11)	---	−7.4*
SP(4–11)	13.42	−2.5*
SP(5–11)	13.50	−2.7*
[Thi^8^,Met(O_2_)^11^]SP(5–11)	12.32	−4.7*
[^99m^Tc]NS_3_/CN-SP(1–11), **1a**	15.95	0.26 ± 0.05
[^99m^Tc]NS_3_/CN-[Thi^8^,Met(O_2_)^11^]SP(1–11), **1b**	---	---
[^99m^Tc]NS_3_/CN-SP(4–11), **1c**	17.53	0.09 ± 0.02
[^99m^Tc]NS_3_/CN-SP(5–11), **1d**	18.41	1.14 ± 0.01
[^99m^Tc]NS_3_/CN-[Thi^8^,Met(O_2_)^11^]SP(5–11), **1e**	17.06	0.64 ± 0.02

*values estimated using the program MarvinSketch, ver. 18.17.0 ChemAxon, (Budapest, Hungary). Values were determined theoretically and indicate only the direction of the changes in lipophilicity of SP fragments.

**Table 2:** Physico-chemical properties of [^177^Lu]DOTA-SP radiobioconjugates (R_T_ values were determined using HPLC (system 2)).

Compounds	R_T_ (min)	log*P*	Stability in Human Serum: % of Intact Radiobioconjugate After			
			1 h	2 h	4 h	24 h
[^177^Lu]DOTA-SP(1–11), **2a**	12.53	−5.12 ± 0.06	100	100	100	72.2
[^177^Lu]DOTA-[Thi^8^,Met(O_2_)^11^]SP(1–11), **2b**	11.67	−5.28 ± 0.02	97.7	100	100	74.8
[^177^Lu]DOTA-SP(4–11), **2c**	13.13	−2.93 ± 0.01	100	92.1	81.0	17.2
[^177^Lu]DOTA- SP(5–11), **2d**	13.23	−2.68 ± 0.01	18.6	8.3	3.6	0
[^177^Lu]DOTA-[Thi^8^,Met(O_2_)^11^]SP(5–11), **2e**	11.97	−3.94 ± 0.01	100	79.2	56.4	4.0

**Table 3:** Biological properties of selected radiobioconjugates.

Radiobioconjugate	*K_d_* [nM]	*B_max_* [nM]
[^177^Lu]DOTA-[Thi^8^,Met(O_2_)^11^]Substance P(1–11), **2b**	11.1 ± 2.7	0.46 ± 0.08
[^177^Lu]DOTA-Substance P(4–11), **2c**	13.3 ± 1.6	1.28 ± 0.22
[^177^Lu]DOTA-[Thi^8^,Met(O_2_)^11^]Substance P(5–11), **2e**	48.7 ± 5.1	0.15 ± 0.05
